# CHIP: A new modulator of human malignant disorders

**DOI:** 10.18632/oncotarget.8219

**Published:** 2016-03-20

**Authors:** Zhe Cao, Guanqiao Li, Qianqian Shao, Gang Yang, Lianfang Zheng, Taiping Zhang, Yupei Zhao

**Affiliations:** ^1^ Department of General Surgery, Peking Union Medical College Hospital, Chinese Academy of Medical Sciences and Peking Union Medical College, Beijing, China; ^2^ Department of Nuclear Medicine, Peking Union Medical Hospital, Chinese Academy of Medical Sciences and Peking Union Medical College, Beijing, China

**Keywords:** CHIP, cancer, co-chaperon, ubiquitination

## Abstract

Carboxyl terminus of Hsc70-interacting protein (CHIP) is known as a chaperone-associated E3 for a variety of protein substrates. It acts as a link between molecular chaperones and ubiquitin–proteasome system. Involved in the process of protein clearance, CHIP plays a critical role in maintaining protein homeostasis in diverse conditions. Here, we provide a comprehensive review of our current understanding of CHIP and summarize recent advances in CHIP biology, with a focus on CHIP in the setting of malignancies.

## INTRODUCTION

Protein is the fundamental entity in the maintenance of cellular functions [1]. Optimum protein performance is highly dependent on the network of molecular chaperones, which are constituents of protein quality control system [2]. The chaperones function to check and facilitate the refolding of misfolded polypeptides, thus responsible for degradation of ubiquitin-tagged proteins [3].

Carboxyl terminus of heat shock cognate protein 70 (Hsc70)-interacting protein (CHIP), a co-chaperone E3 ligase, was initially identified as a novel tetratricopeptide repeat (TPR)-containing protein in 1999 [4]. It is also known as STIP1 homology and U-box containing protein 1 (STUB1). This gene encodes a novel 34.5-kDa cytoplasmic protein with a deduced amino acid sequence of 303 residues. The expression of CHIP is ubiquitously but varies among tissues. For example, hypermetabolic tissues such as skeletal muscle and heart have high expression. As CHIP is an evolutionarily ancient protein, *chip* gene shows extensive phylogenetic conservation [4–6]. Human CHIP exhibits 98% amino acid similarity with mouse CHIP and 60% with Drosophila CHIP [1].

As a connecting link between chaperones and ubiquitin proteasomes, CHIP is actively involved in a diverse array of cellular processes such as protein refolding, degradation, protein trafficking, signaling, transcription and apoptosis [7]. Hence, it is not surprising that CHIP regulates abundant proteins and consequently ailments which arise due to abnormalities in those proteins. This review provides the current understanding of CHIP and its binding partners, followed by the diverse roles of CHIP in human disorders, with a focus of cancer.

## CHIP STRUCTURE AND FUNCTION

**Figure 1:**
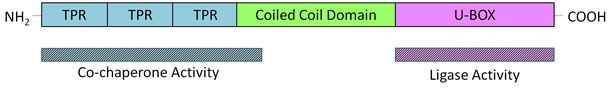
Structure of CHIP, a 34.5-kDa cytoplasmic protein with a deduced amino acid sequence of 303 residue CHIP consists of triple tandem TPR domains, U-box domain and a central coiled coil domain.

CHIP comprises triple tandem TPR domains, a U-box domain and a central coiled coil domain (Figure 1) [8]. The 34-amino-acid TPR domains at the amino terminus with an adjacent charged region (amino acid residues 1-197) together form a binding site for heat shock protein (Hsp)/Hsc70 and Hsp90. Notably, the recruitment of Hsp/Hsc70 by CHIP involves a reciprocal allosteric interaction between the TPR and U-box domains [9]. Such an interaction stimulates numerous biochemical reactions and subsequent physiological functions. For instance, CHIP and Hsp90 heterocomplex elicits release of the regulatory cofactor p23, thereby suppressing the affinity and refolding activity of Hsp90 for substrate proteins [5,10,11]. On the other hand, CHIP competes for Hsp70 from Hsp40/Hsp70 complex, which attenuates their ATPase activity and refolding capacity for denatured substrates [4]. Consequently, due to inhibition of accompanying Hsp, CHIP acts as a bridge between chaperones and the proteasome system; that is, CHIP transforms the refolding machinery into the destructive pathway [12].

At the carboxyl terminus, the U-box domain contributes to its ligase activity [13]. Of note, CHIP is the first identified chaperone that possesses intrinsic E3 ubiquitin ligase activity. General E3 ubiquitin ligases contain a Homologous to E6AP Carboxyl Terminus (HECT) or Really Interesting New Gene (RING) domain. U-box domain in CHIP is structurally and functionally similar to the RING domain [13]. The U-box domain can bind to UbcH4/UbcH5 and deliver ubiquitin molecule from E2 to an unfolded substrate protein, whereas CHIP (ΔU) lacking the U-box domain cannot [6]. Between the TPR and U-box domains, the central domain is rich in charged residues, with two possible nuclear localization signals. This charged domain might facilitate TPR-dependent interactions [1].

Although the chaperone functions of CHIP have been well characterized over the past decades, the underling mechanisms of proteasomal degradation remain largely unclear. S5a and HC8 proteasome subunits [5] or Bcl-2-associated athanogene 1 (BAG-1) [14] have been found to facilitate cooperation between CHIP and proteasome. Particularly, BAG-1 binds to proteasome via its carboxy-terminal BAG domain, thus targeting chaperone substrates for degradation [14]. BAG-2, however, inhibits the ubiquitin ligase activity by abrogating the CHIP/E2 interaction [15].

## CHIP INTERACTION WITH FUNCTIONAL PROTEIN

### Interactions between CHIP and heat shock protein

Recent advances in cellular biology and biochemistry have led to widespread acceptance of the concept that CHIP is a bona fide binding partner to diverse proteins, of which Hsp is the major one. Similar to CHIP, Hsp is highly conserved across species and widely distributed among plant and animal cells. Any stress, including heat, hypoxia, as well as cancer, can induce Hsp production. Normally, it functions as chaperone to participate in refolding of vital cellular proteins, which promotes cellular proliferation and inhibits apoptosis. For example, Hsp70 increases the expression of anti-apoptotic protein Bcl-2, further protecting neurons and astrocytes from anoxic conditions [16, 17].

Previous studies have demonstrated that Hsp70-/Hsp90-dependent chaperone machinery is required for CHIP activity. Interestingly, Hsp90 stabilizes associated client proteins, whereas Hsp70 promotes protein degradation by polyubiquitination [18]. Via this mechanism, CHIP ubiquitinates and degrades glucocorticoid receptor (GR), androgen receptor (AR), estrogen receptor (ER), ErbB2, and α-synuclein, only when bound to Hsp [19–21]. Theoretically, all of the substrates of Hsp70 or Hsp90 are potential substrates of CHIP. On the other hand, CHIP can directly bind to specific substrates independent of Hsp. Rosser MF *et al.* [22] demonstrated that, CHIP bound to certain proteins (such as Smad1, HSF, Raf-1) through intrinsic polypeptide binding sites, indicating CHIP itself is a molecular chaperone upon stress [23]. Interestingly, CHIP plays a dual role in regulation of Hsp70 expression. In the early stage of stress, CHIP increases heat shock factor 1 (HSF1) activity, thereby enhancing Hsp70 expression and inhibiting apoptosis [24]. In the recovery stage, however, CHIP leads to ubiquitination and degradation of the Hsp70-bound denatured protein as well as of Hsp70 [25]. This dual-phase modulation allows for cellular homeostasis.

### Interaction between CHIP and membrane receptors

**Her2/ErbB2**. Her2/ErbB2, a member of the epidermal growth factor receptor (EGFR) family, is a transmembrane protein tyrosine kinase. As an oncogene, phosphorylated Her2 triggers activation of various signaling pathways involved in cell proliferation and tumorigenesis. Her2 is overexpressed in 30% of breast cancer and correlates with disease recurrence and prognosis [26]. In the presence of Hsp90, CHIP ubiquitylates Her2, decreasing its endogenous expression. Moreover, CHIP regulates Hsp90 inhibitor geldanamycin (GA) to promote Her2 degradation [27].

**c-Met**. The tyrosine kinase receptor c-Met (also known as hepatocyte growth factor receptor or HGFR) is overexpressed in a variety of human cancers [28]. Given that c-Met activates cellular growth, migration and invasion, its aberrant expression correlates with poor patient prognosis. In the case of lung cancer, CHIP overexpression destabilizes endogenous c-Met and inhibits cancer growth and invasion, whereas CHIP knockdown increases c-Met expression, suggesting a critical effect of CHIP in c-Met degradation [29].

**Hormone receptors**. Glucocorticoid receptor (GR), a member of a large superfamily of nuclear receptor, modulates genes controlling development, metabolism, and immune response. CHIP inhibits GR-mediated signal transduction via dissociation of GR from Hsp90. Besides, CHIP promotes ubiquitination of GR in collaboration with Ubc13 [30]. In the setting of androgen receptor (AR), CHIP interacts with the androgen receptor NH2-terminal conserved motif in a phosphorylated specific site [30], which triggers ubiquitination and subsequent degradation [31]. Since AR plays an essential role in the onset and progression of prostate cancer, AR degradation leads to mitotic arrest in prostate cancer cells treated with low dose 2-methoxyestradiol (2-ME, an endogenous estrogen metabolite) [32]. Interestingly, the degradation is considered to be ubiquitin-dependent, mediated by CHIP. Furthermore, CHIP attenuates the monomeric mutant AR in spinal and bulbar muscular atrophy (SBMA) more effectively than does wild type AR, indicating that mutant AR or other substrates are more sensitive to CHIP [33]. Estrogen receptor α (ERα) is another substrate of Hsp90. CHIP induces ERα degradation via direct ubiquitination [34] or the histone deacetylase inhibitor suberoylanilide hydroxamic acid (SAHA) [35], thereby blocking survival signaling in breast cancer cells.

### Interaction between CHIP and signaling proteins

**PI3K/AKT**. The PI3K/AKT pathway is one of the most important signaling pathways in the regulation of cellular growth. Aberrant activation contributes to tumor growth and angiogenesis in diverse tumors [36]. CHIP forms a complex with Hsp90 and AKT. Once this triple complex is assembled, the ubiquitination and degradation of phosphorylated AKT occurs, thereby shutting down the over-active machinery and maintaining cellular pathway homeostasis [37]. Hsp70/CHIP complex is a novel E3 ligase for the p85 subunit of phosphatidylinositol 3-kinase (PI3K) which is responsible for p85 ubiquitination and degradation. Phosphatases, in addition to kinases, are another category of CHIP targets. Phosphatase and tensin homolog (PTEN), the first identified tumor suppressor possessing phosphatase activity, dephosphorylates the target molecule phosphatidylinositol 3,4,5-trisphosphate (PI(3,4,5)P3) downstream of (PI3K to generate PI (4, 5)P2. Thus, PTEN blocks the activation of downstream AKT. In prostatic carcinoma cells, transient binding to the TPR domain of CHIP leads to PTEN ubiquitination and subsequent degradation by the proteasome system [38]. Furthermore, CHIP promotes NF-κB ubiquitination and degradation in gastric cancer cells. NF-κB protein, via activation in the downstream cascade of the PI3K/AKT pathway, is involved in versatile expression of interleukins and chemokines. Additionally, CHIP allows NF-κB to enter the nucleus, where it functions as a transcription factor and suppresses the expression of its target genes (such as IL-8, MMP-2, VEGF). In this way, CHIP inhibits the invasive and metastatic capacities as well as the angiogenic ability of gastric cancer [39].

**MAPK**. Mitogen-activated protein kinases (MAPKs) comprise a family of cellular serine/threonine protein kinases that control embryogenesis, cell differentiation, proliferation and death [40]. Apoptosis signal-regulating kinase 1 (ASK1), a member of MAP3K family, plays an important role in the pathogenesis of neurodegeneration, cardiovascular diseases and tumorigenesis [41]. Under oxidative stress or inflammatory factor-mediated activation, phosphorylated ASK1 activates the downstream c-Jun N-terminal kinase (JNK) and p38 MAPK pathways, thereby inducing cellular apoptosis. The C-terminus of Hsp70 has affinity for the acceptor domain at the N-terminal TPR of ASK1. Through cooperation with CHIP, Hsp70 promotes the ubiquitin-dependent proteasomal degradation of ASK1 and inhibits TNF-alpha-induced cell apoptosis [42]. In Addition, CHIP triggers translocation of the ASK1 partner death domain-associated protein (Daxx) into the nucleus, further initiating an anti-apoptotic response [43].

**p53**. P53 is the first characterized and most popular tumor suppressor gene in almost all cancer types. DNA-bound p53 recruits DNA repair proteins and induces mitotic arrest from G1 to S phase. Hence, p53 acts as a guard of the genome against cancerogenesis. Cellular accumulation of mutant p53, on the other hand, induces tumor formation and transformation [44]. MDM2, the main ubiquitin ligase of p53, plays a pivotal role in stimulating p53 turnover via ubiquitin-dependent proteasomal degradation [45]. Interestingly, in normal conditions, CHIP degrades wild-type p53 protein alone, but only targets and ubiquitinates mutant p53 in the presence of Hsp70 or Hsp90 [46]. Upon heat stress, however, CHIP directly interacts with wild-type p53 as a chaperone and inhibits its denaturation. Together with Hsp90 inhibitors, CHIP preferentially binds to misfolded p53 and restores its DNA binding activity [47]. Collectively, CHIP might serve as a chaperone of wild-type p53. It maintains its conformation under physiological condition and rescues mutant p53 into its native, folded state under stress. Furthermore, CHIP assists in degradation of p53 isoform 133p53α degradation, which orchestrates p53-mediated cellular senescence [48]. As a consequence, CHIP functions as a critical modulator with multifaceted effects on the expression and function of p53.

**TGF-β**. The transforming growth factor-β (TGF-β) pathway regulates cell differentiation, apoptosis and migration by conducting signaling into the nucleus and inducing gene expression through the Smad [49] or Daxx signaling pathway [50]. CHIP with TPR domain binds to the C-terminus of Smad1/5, leading to Smad1/5 ubiquitination and degradation. The phosphorylated SXS motif at the C-terminus of Smad1 enhances its binding affinity for CHIP. Hsp70 and Hsp90 play opposing effects of regulating TGF-β signaling via CHIP-mediated Smad3 ubiquitination and degradation [51]. Additionally, Daxx, an important apoptosis-associated factor, synergizes with the Axin/HIPK2/p53 complex to induce programmed cell death [52]. The stress-induced association of CHIP with Daxx forms a special insoluble fragment and avoids degradation by the proteasome. At the meantime, it inhibits stress-induced p53-dependent apoptosis by interfering with interaction between Daxx and homeodomain-interacting protein kinase 2 (HIPK2). Once the stress is eliminated, Daxx transforms to the soluble functional compartment [53].

**TLR**. Toll-like receptors (TLRs), expressed mainly on antigen-presenting cells (APCs), is pivotal in bridging innate and adaptive immunity. By recognizing structurally conserved pathogen components termed pathogen-associated molecular patterns (PAMPs) and/or endogenous damage-associated molecular patterns (DAMPs), TLRs trigger cellular signaling pathways and immune responses. In the TLR-induced signaling cascade, CHIP interacts with and polyubiquitinates downstream Src (non-receptor tyrosine kinase) and PKCζ, thereby facilitating LPS/CpG-induced Src and PKC recruitment and activation [54]. The activated kinases further induce the nuclear translocation of NF-κB and IRF3/7, leading to release of cytokines such as IL-6 and IFN-β.

## ROLE OF CHIP IN HUMAN DISEASES

Given its functions as a quality control E3 ligase, CHIP is pivotal in regulating myriad of proteins and pathologies with abnormal protein expression. Accumulating evidence demonstrates the importance of CHIP in cancer, neurological disorders, cardiac disease and so forth.

### CHIP and cancer

**Figure 2:**
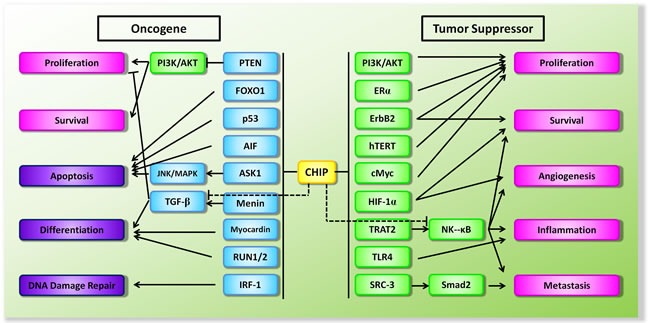
Schematic diagram of the dual functions of CHIP in the context of tumorigenesis Proteins in square are either oncogenes in green or tumor suppressor genes in blue. Arrow (−>) indicates promotion and the symbol (−I) indicates suppression.

Cancer remains the most frequently studied disease in CHIP biology due to aberrant expression of diverse protein in the progression of cancer [55]. Notably, since controversy regarding the oncogenic or tumor suppressive effect of CHIP emerged over the past several years, CHIP has been considered as a double-edged sword in modulation of tumorigenesis (Figure 2).

### CHIP as a tumor suppressor

Since Kajiro et al. [56] demonstrated the tumor suppressive function of CHIP as a hallmark, a series of subsequent studies have elucidated the critical roles of CHIP in proliferation, tumorigenesis and invasion in several malignancies. In the pioneering study, Kajiro et al. observed tumor growth, metastasis and malignancy were inversely correlated with CHIP levels in a nude mouse xenograft human breast tumor model [56]. Conversely, CHIP knockdown (shCHIP) in cancer cells readily resulted in tumor development and a metastatic phenotype in mice. The suppression of tumorigenesis might result from the inhibition of breast cancer stem cells [57]. Likewise, in human breast cancer specimens, CHIP mRNA expression was negatively associated with the malignant grade (TNM stage) and overall patient survival [58]. Collectively, CHIP might be a potentially favorable prognostic marker for breast cancer.

Recently, CHIP has been documented as a tumor suppressor in a variety of settings, with emerging evidence of myriad protein targeted by CHIP (Table 1). Our previous work demonstrated that CHIP ubiquitinated EGFR for proteasome-mediated degradation in pancreatic cancer cells and suppressed EGFR downstream signaling pathways [59]. Consistent with the findings in breast cancer, decreased CHIP expression in pancreatic cancer tissues correlated with poor prognosis and shortened overall survival of patients. In accordance with our observation, a negative correlation has been identified between CHIP expression and tumor malignancy (e.g. TNM stage and lymph node metastasis) in human gastric cancer [39, 60]. Unlike targeting EGFR in pancreatic carcinoma, CHIP bound to NF-κB/p65 and triggered its ubiquitination and proteasomal degradation, which further inhibits NF-κB signaling and IL-8-induced angiogenesis. Therefore, decreased CHIP expression was associated with tumor metastasis, and served as an independent prognostic factor for patients with gastric cancer [39]. The suppressive role is further confirmed in colorectal cancer, with evidence that CHIP overexpression led to decreased expression of NF-κB-targeted oncogenes [61]. Chronic myeloid leukemia (CML) is a non-solid neoplasm that is genetically characterized by the BCR-ABL fusion protein. Not surprisingly, CHIP interacts with the immature fused protein and induces its ubiquitin-dependent degradation, thereby hindering its proliferative ability [62]. In the setting of prostate cancer, AR is a target of CHIP-mediated degradation [32]. As discussed above, the destruction of AR blocks mitosis and further limits tumor proliferation. These findings shed light on the possibility of targeting E3 ligases such as CHIP as a novel strategy to attenuate AR expression and limit prostate cancer.

**Table 1:** Clinial studies/Human cancer cell studies regarding CHIP and cancer

Cancer Type	CHIP expression in human cancer	Clinical Functions	Cellular Functions	Target/Substrate	Reference
Breast Ca.	↓	(+)Malignancy	Suppression of tumour growth and metastasis	SRC-3	Kajiro et al(2009) [56]
Breast Ca.	↓	(−) TNM Stage, (+)OS	-	-	Patani et al(2010) [58]
Gastric Ca.	↓	(−) TNM Stage, LN Metastasis	-	-	Gan et al(2012) [60]
Gastric Ca.	↓	(−) Metastasis	Inhibition of blood vessel formation and growth of xenografts in nude mice	NF-kB/p65	Wang S et al(2013) [39]
Pancreatic Ca.	↓	(+)Prognosis, (+)OS	Suppression of cell proliferation,anchor-independent growth, invasion and migration	EGFR	Wang T et al(2014) [59]
Colorectal Ca.	↓	-	Inhibition of tumor growth, migration and invasion	NF-kB	Wang Y et al (2014) [61]
Colorectal Ca.	↓	-	-	eIF5A	Shang et al (2014) [63]
Prostate Ca.	-	-	Mitotic arrest	AR	Sarkar(2014) [32]
Gallbladder Ca.	↑	(−)Prognosis, (−)OS	-	-	Liang et al(2013) [72]
Esophageal Ca.	↑	(+)Metastatic LNs	-	-	Wen et al(2013) [73]
Prostate Ca.	↑	-	Regulation of PTEN-dependent transcription	PTEN	Ahmed et al.(2012) [38]

(+): Positive Correlation, (−): Negative Correlation, Ca.: Cancer, OS: Overall Survival, LN:Lymph Node, ↑:up-regulation, ↓:down-regulation

Thus far, a variety of oncogenic substrates have been identified, including ErbB2, eIF5A, SRC-3, pAkt, c-Myc and hypoxia-inducible factor 1α (HIF-1α), in different types of cancer [27, 56, 63–67]. Notably, K63 ubiquitination mediated by CHIP is necessary for HIF-1α degradation by chaperone-mediated autophagy [68]. These observations indicate a profound and versatile protein network regulated by CHIP in the control of tumorigenesis, and thus CHIP might be an upstream target for therapeutic intervention.

### CHIP as an oncogene

In contrast to investigations mentioned above, a number of publications have reported CHIP to target tumor suppressor genes (TSGs), acting as an oncogene (Table 1). Li et al. [69] observed CHIP with the function of ubiquitination and degradation of Forkhead box (Fox) containing transcription factor O1 (FoxO1) in response to tumor necrosis factor-α (TNF-α) stimulation. Given that FoxO1 is responsible for apoptosis, CHIP assists in survival and proliferation of smooth muscle cells following TNF-α induction. Consistently, in prostate cancer cells [38], expression of PTEN correlated inversely with CHIP. Furthermore, CHIP has also been implicated in the modulation of other TSG proteins, including apoptosis-related p53, apoptosis-inducing factor (AIF), and interferon regulatory factor 1 (IRF-1) [46, 70, 71].

Interestingly, Liang et al. [72] reported higher CHIP expression assessed by immunohistochemistry among patients with shorter gallbladder cancer specific survival. This observation suggests a correlation between CHIP expression and poor prognosis in gallbladder carcinoma (GBC). CHIP levels, however, were not associated with other common clinicopathological elements, such as TNM stage. Likewise, significantly high level of CHIP was found in 163 metastatic lymph nodes (MLNs) of esophageal squamous cell carcinoma (ESCC), whereas the primary tumor of ESCC shared comparable level with normal epithelium [73]. Additionally, relatively lower expression of CHIP in MLNs correlated with better survival. Hence, CHIP possibly serves as an independent prognostic factor in ESCC.

The studies mentioned above were limited by their relatively small clinical sample size and retrospective design; these limitations could in part explain the varied results. Despite all the variations, CHIP obviously regulates various aspects of tumors in an intricate and exquisite manner (Figure 2). Apparently, further clarification is of great necessity to elucidate its pathogenic mechanisms in human malignancy.

## CHIP AND OTHER DISEASES

### CHIP and neurological disorders

**Figure 3:**
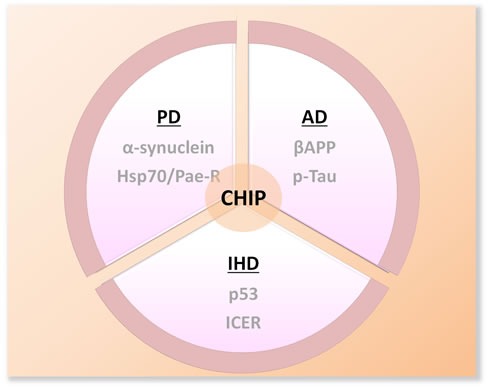
Schematic diagram of the substrates of CHIP in several well-studied neruodegenerative disorders PD:Parkinson's Disease; AD: Alzheimer's Disease; IHD: Ischemic Heart Disease; Hsp70: heat shock protein 70; Pael-R:Parkin-associated endothelin-like receptor; βAPP:β-amyloid precursor protein; p-Tau: phosphorylated Tau; ICER: inducible cAMP early repressor.

CHIP has been extensively linked to diverse neurological diseases, largely due to its capacity to remove aberrant disease-causing proteins (Figure 3). In particular, CHIP plays a vital role in the pathogenesis of neurodegenerative diseases (such as Parkinson's disease and Alzheimer's disease) that involve accumulation of toxic protein aggregates with aging.

Parkinson's disease. Parkinson's disease (PD) is characterized by preferential loss of dopaminergic neurons in the substantia nigra and the accumulation of Lewy bodies (LB), containing α-Synuclein protein [74–75]. CHIP co-localizes with α-synuclein and inhibits its aggregation via proteasomal degradation and suppression of oligomerization [76, 77]. In addition, Parkin, a RING-finger type E3 ligase, is responsible for the proper disposal of multiple substrates involved in PD, including mutated a-synuclein, glycosylated synuclein-22, Parkin-associated endothelin-like receptor (Pael-R), synphilin, cell division control-related protein and a polyglutamine-expanded ataxin-3 mutant [19, 78, 79]. Interestingly, the bifunctional co-chaperone CHIP aids in the dissociation of Hsp70 from the Pael-R/Parkin complex, and this displacement is necessary for Parkin to resume E3 activity toward Pael-R [80].

Alzheimer's disease. Alzheimer's disease (AD) is characterized by accumulation of misfolded insoluble β-amyloid and hyperphosphorylated Tau protein [81]. As a chaeperone, CHIP prevents normal β-amyloid precursor protein (βAPP) from degradation. On the other hand, CHIP, as a ligase, cooperates with Hsp and promotes βAPP degradation in an ubiquitin-dependent manner [82]. In this way, the progression of AD is hindered due to less accumulation of β-amyloidin neurons. In the setting of Tau protein, it serves to stabilize microtubules in neurons, but the phosphorylated form is prone to form toxic neurofibrillary tangles [83]. With the functions of eliminating unwanted protein aggregation, CHIP together with Hsp70 pushes p-Tau towards proteasomal degradation by targeting the microtubule-binding repeat region of hyperphosphorylated tau [84–86].

### CHIP and cardiac diseases

Apoptosis plays a critical role in cardiac remodeling in the setting of ischemic heart injury (Figure 3) [87]. Targeted ablation of p53 suppresses apoptosis and concomitant cardiac rupture after myocardial infarction (MI) in mice [89]. In the screening of heart cDNA pools, CHIP has been identified as an endogenous p53 antagonist in the heart [89]. CHIP decreased p53 level via ubiquitination and proteasomal degradation. Furthermore, Woo et al., [90] found that the cardioprotective function of CHIP-ERK5 (extracellular signal-regulated kinase 5) interaction through destabilization of inducible cAMP early repressor (ICER). In contrast, dissociation of the complex via phosphorylation at S496 of ERK5 results to reversal of cardioprotective phenotype [91].

## CONCLUSION

The chaperone and ubiquitin-proteasome systems are the two most important mechanisms that work cooperatively to maintain protein quality control and cellular homeostasis. As a linker between these two systems, CHIP plays a pivotal role in a diverse number of cellular processes involving protein refolding, degradation, trafficking, apoptosis, inflammation and morphogenesis in both physiological and pathological settings. Increasing evidence suggests that CHIP is critical in cancers, neurological disorders, and cardiac diseases, among others [7,55]. Despite some discrepancies, CHIP generally exerts a cyto-protective effect by eliminating detrimental forms of several proteins.

However, many questions remain to be answered, including the underlying mechanism of target selection and the types of polyubiquitin chains as well as the manner by which signal recognition and the transfer of substrates occur in the proteasome system. Further explorations into the regulation and functional aspects of CHIP will provide deeper and more fundamental insights into the biological roles of CHIP and its therapeutic potential for various diseases.
